# Trends in multimorbidity and polypharmacy in the Flemish-Belgian population between 2000 and 2015

**DOI:** 10.1371/journal.pone.0212046

**Published:** 2019-02-12

**Authors:** Marjan van den Akker, Bert Vaes, Geert Goderis, Gijs Van Pottelbergh, Tine De Burghgraeve, Séverine Henrard

**Affiliations:** 1 Department of Family Medicine, School Caphri, Maastricht University, Maastricht, the Netherlands; 2 Department of Public Health and Primary Care, University of Leuven (KU Leuven), Leuven, Belgium; 3 Louvain Drug Research Institute and Institute of Health and Society (IRSS), Université catholique de Louvain (UCL), Brussels, Belgium; Sciensano, BELGIUM

## Abstract

**Objectives:**

The aim of this paper was to describe the time trends in the prevalence of multimorbidity and polypharmacy in Flanders (Belgium) between 2000 and 2015, while controlling for age and sex.

**Methods:**

Data were available from Intego, a Flemish-Belgian general practice-based morbidity registration network. The practice population between 2000 and 2015 was used as the denominator, representing a mean of 159,946 people per year. Age and gender-standardised prevalence rates were used for the trends of multimorbidity and polypharmacy in the total population and for subgroups. Joinpoint regression analyses were used to analyse the time trends and breaks in trends, for the entire population as well as for specific age and sex groups.

**Results:**

Overall, in 2015, 22.7% of the population had multimorbidity, while the overall prevalence of polypharmacy was 20%. Throughout the study period the standardised prevalence rate of multimorbidity rose for both sexes and in all age groups. The largest relative increase in multimorbidity was observed in the younger age groups (up to the age of 50 years). The prevalence of polypharmacy showed a significant increase between 2000 and 2015 for all age groups except the youngest (0–25 years).

**Conclusion:**

For all adult age groups multimorbidity and polypharmacy are frequent, dynamic over time and increasing. This asks for both epidemiological and interventional studies to improve the management of the resulting complex care.

## Introduction

Multimorbidity—the co-occurrence of two or more chronic diseases in a patient [1] and polypharmacy—the prescription of five or more medications in one year [2] are broadly recognized as important and interrelated phenomena [3].

The consequences of multimorbidity have often been studied and have been reported on an aggregated level, with even a recent overview of systematic reviews [4]. In his review McPhail reported a curvilinear, near exponential association between additional chronic diseases and health care costs [5]. Fortin and colleagues reviewed original studies of the quality of life in patients with multimorbidity; despite methodological shortcomings and the diversity of the studies, they reported a clear inverse relation [6]. There was also a diverse picture for mortality, but an overall increased risk of death among patients with multimorbidity was reported [7].

Traditionally, multimorbidity research has focussed on older people and predicts an alarming picture of future developments [8]. However, in absolute numbers, the majority of people with multimorbidity are still under 65 years of age [9, 10].

Polypharmacy is frequently found among people with multimorbidity: the disease number is a stronger predictor for the number of medications prescribed than age is [11]. Like multimorbidity, polypharmacy is famous for its negative consequences, such as diminished adherence and more frequent adverse events. Approximately 6.5% of all emergency hospital admissions are attributable to adverse drug events, and at least half of these are judged to have been preventable [12, 13].

Estimating the additional health care costs of multimorbidity and polypharmacy is not straightforward, as some combinations result in a synergetic cost effect and some have shown a disproportionate impact on health care utilization far beyond the simple addition of costs [5]. A recent overview revealed that multimorbidity is related to higher health care costs, not only for emergency hospital admissions, but also for more frequent visits to primary care and hospital specialists, more hospital admissions and a higher number of bed days in hospital, and more medication use [5].

Multimorbidity and polypharmacy are obviously closely related. The prescription of appropriate medication, balancing harm and benefit and following medical guidelines, becomes increasingly difficult with a growing number of chronic medical conditions [14]. Among older patients with multimorbidity and polypharmacy, this balance is even more fragile, due to ageing-related changes such as decreased liver and kidney function, more sensitive receptors and decreased homeostatic reserves.

It is often reported that the number of people suffering from chronic diseases, multimorbidity and polypharmacy has increased in the past decades. This is mainly based on cross-sectional studies over time, in different populations [15]. Time trends in the prevalence of multimorbidity and polypharmacy are scarce [16–18]. The Flemish primary care-based Intego network offers an excellent opportunity to evaluate those trends.

The aim of this paper is to describe the time trends in the prevalence of multimorbidity and polypharmacy between 2000 and 2015 in Flanders (Belgium) while controlling for age and sex.

## Materials and methods

### Data source

Data were available from Intego, a Flemish-Belgian general practice-based morbidity registration network at the Academic Centre of General Practice of the KU Leuven [19]. Around 100 general practitioners (GPs) provide annual information about all their patients through a trusted third party. Collaborating GP practices are spread over Flanders (Belgium). Before GPs are accepted as a participant in Intego, they have to fulfil three quality criteria. First, the average number of new diagnoses per patient per year should be higher than one. Second, diagnoses have to be entered in the practice software using keywords. Diagnoses are automatically classified using an extensive thesaurus, which translates keywords into the International Classification of Primary Care (ICPC-2) in the process of data extraction. The percentage of diagnoses recorded without using keywords should be less than 5%. Finally, these parameters must remain stable for at least three years [19]. Data are collected in a routine manner as part of daily practice and contain all new diagnoses together with new drug prescriptions, as well as laboratory test results and some background information (including gender and year of birth). Registered data are continuously updated and historically accumulated for each patient. For medication, the Anatomical Therapeutic Chemical (ATC) classification system is used.

### Study population

In the present study, data available from 31 December 2015 were used. The practice population, as calculated from all people in the yearly contact groups in Intego between 2000 and 2015, was used as the denominator [20]. This represented a mean of 159,946 people in the practice population per year, varying between 115,328 and 186,829 people (see S1 Table for the exact numbers per year). Throughout the study period 79 practices provided their data, with 73% contributing for 13 or more years (see S1 Fig for more detailed information).

### Measures

For this study, multimorbidity was defined as the co-occurrence of two or more chronic diseases in a patient [1, 4]. For the assessment of multimorbidity the year-prevalence of the diseases was used. An overview of the chronic diseases considered in this study is presented in S2 Table [21]. Polypharmacy was defined as the prescription of five or more different medications in one year [2]. To count medication, the first five characters of the ATC codes were used (ATC level 4).

### Statistical analyses

Age- and gender-standardised prevalence rates were used for the trends of multimorbidity and polypharmacy in the total population and for subgroups. Standardised rates were computed using 5-year age groups based on the distribution of the Flemish-Belgian population in 2015. There were four age groups: 0–24 years, 25–49 years, 50–74 years, and 75 years and older.

Joinpoint regression analyses were used to analyse the time trends in multimorbidity and polypharmacy and breaks in trends, for the entire population, as well as for specific age and sex groups [22]. Joinpoint regression allows identifying periods with a significant change in the trend, and in addition annual percentage change (APC) per time period and average APC over the whole period are computed. Our analyses covered the time window between 2000 and 2015, with the dependent variable being the proportion of people with multimorbidity or polypharmacy, respectively. Trends over a specific period of time were described by the annual percent change (APC), while trends over the whole 2000–2015 period were summarised using the average annual percent change (AAPC). Joinpoint regression models were performed using the Joinpoint Regression Program, Version 4.3.1.0 (Statistical Research and Applications Branch, National Cancer Institute). All other analyses were performed using R Software version 3.1.3 [23]. A p-value <0.05 was considered statistically significant.

The Intego procedures were approved by the ethical review board of the Medical School of the Catholic University of Leuven (no ML 1723) and by the Belgian Privacy Commission (no SCSZG/13/079).

## Results

### Population characteristics in 2015

**Fig 1:**
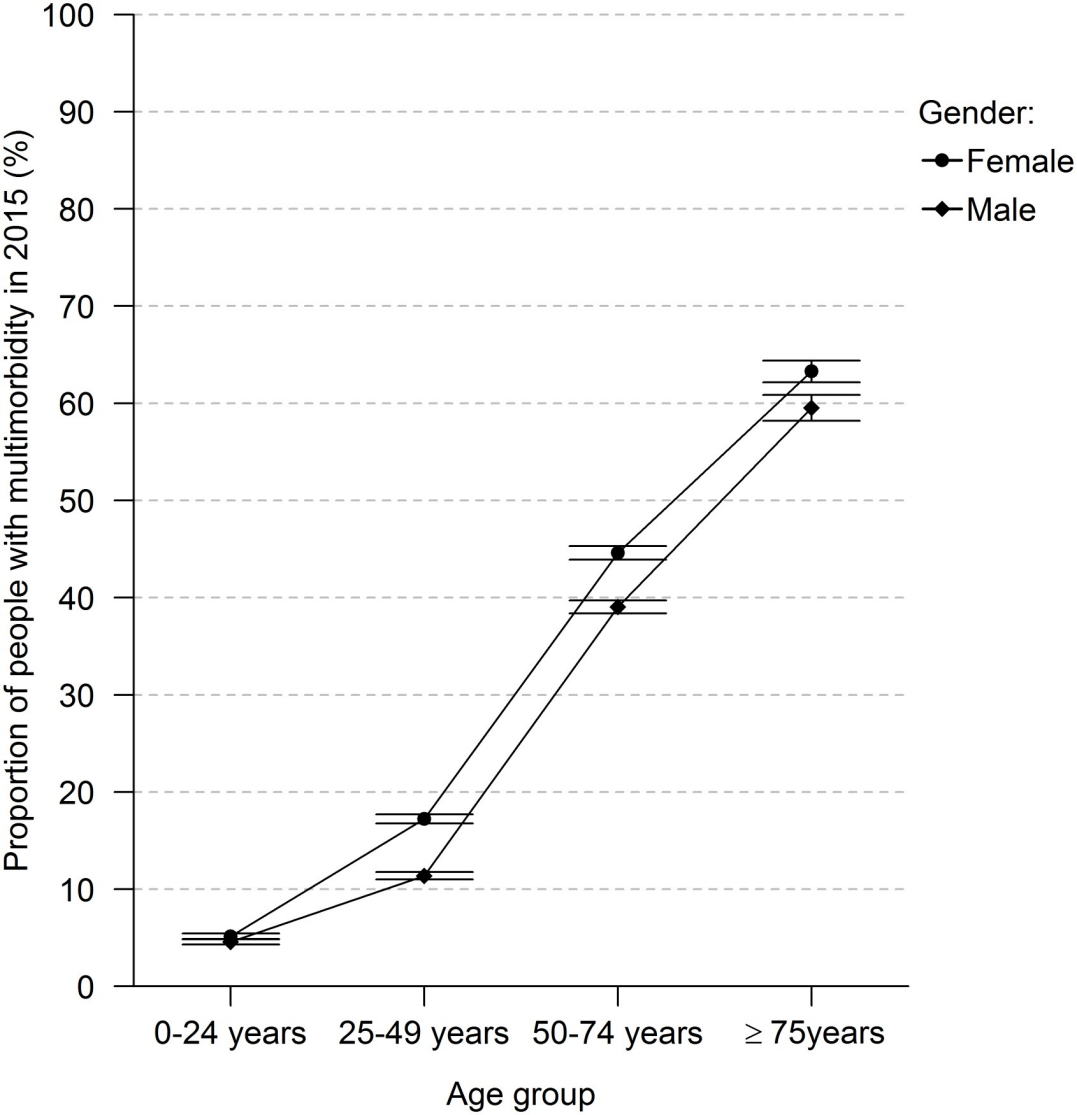
Age trends in the proportions of multimorbidity for males and females (point prevalence, 95% CI).

The practice population totalled 152,270 people in 2015. Of those, 61.5% did not have any chronic disease, ranging from 82.6% in the 0–24 year old group to 25.5% in those aged 75 years and older. Overall, 15.9% had one chronic disease, and 22.7% had multimorbidity. The proportion of both males and females with multimorbidity increased strongly with age, with females having statistically significantly larger proportions with multimorbidity in all age groups (Fig 1 and S3 Table). The *absolute* number of people with multimorbidity was the highest among those aged 50–74 years (N = 16,945), followed by those aged 75 years and older (N = 7,836) and 25–49 years (N = 7,529).

Overall, the prevalence of polypharmacy was 20%. The number of medication prescriptions was also strongly age-related, with just over half of the people aged below 25 years having any medication prescribed to around 3 out of 10 in people aged 75 years and older having no medication prescriptions (28.1%, n = 3565/12,700) (Table 1). The prevalence of polypharmacy ranged from 11.2% in the youngest females to 49.5% in females aged 75 years and older; in males, rates from 8.3% in the youngest to 50.9% in the oldest were found. Here again, we found the highest absolute number in the age group 50–74 (N = 7,008), followed by those aged 25–49 years (N = 3,565), with people of 75+ in third place (N = 2,727).

**Table 1:** Distribution of chronic diseases and medications in 2015, stratified by age groups and sex.

	Females Age groups				Males Age groups			
	0–24 years (N = 22,707)	25–49 years (N = 26,063)	50–74 years (N = 20,044)	≥75 years (N = 7,361)	0–24 years (N = 23,476)	25–49 years (N = 26,742)	50–74 years (N = 20,506)	≥75 years (N = 5371)
Number of chronic diseases								
0	82.3%	63.7%	37.2%	24.9%	83.0%	72.1%	42.9%	26.2%
1	12.6%	19.1%	18.2%	11.8%	12.5%	16.6%	18.0%	14.3%
2–4	5.1%	15.4%	32.1%	35.5%	4.5%	10.6%	30.1%	37.4%
≥5	0.1%	1.9%	12.5%	27.8%	0.1%	0.8%	8.9%	22.2%
Number of medications								
0	48.3%	37.2%	27.0%	28.2%	55.2%	49.4%	32.3%	27.9%
1–4	40.5%	39.8%	31.7%	22.3%	36.5%	37.2%	33.5%	21.2%
≥5	11.2%	23.0%	41.4%	49.5%	8.3%	13.3%	34.2%	50.9%

### Trends in multimorbidity and polypharmacy over time

**Fig 2:**
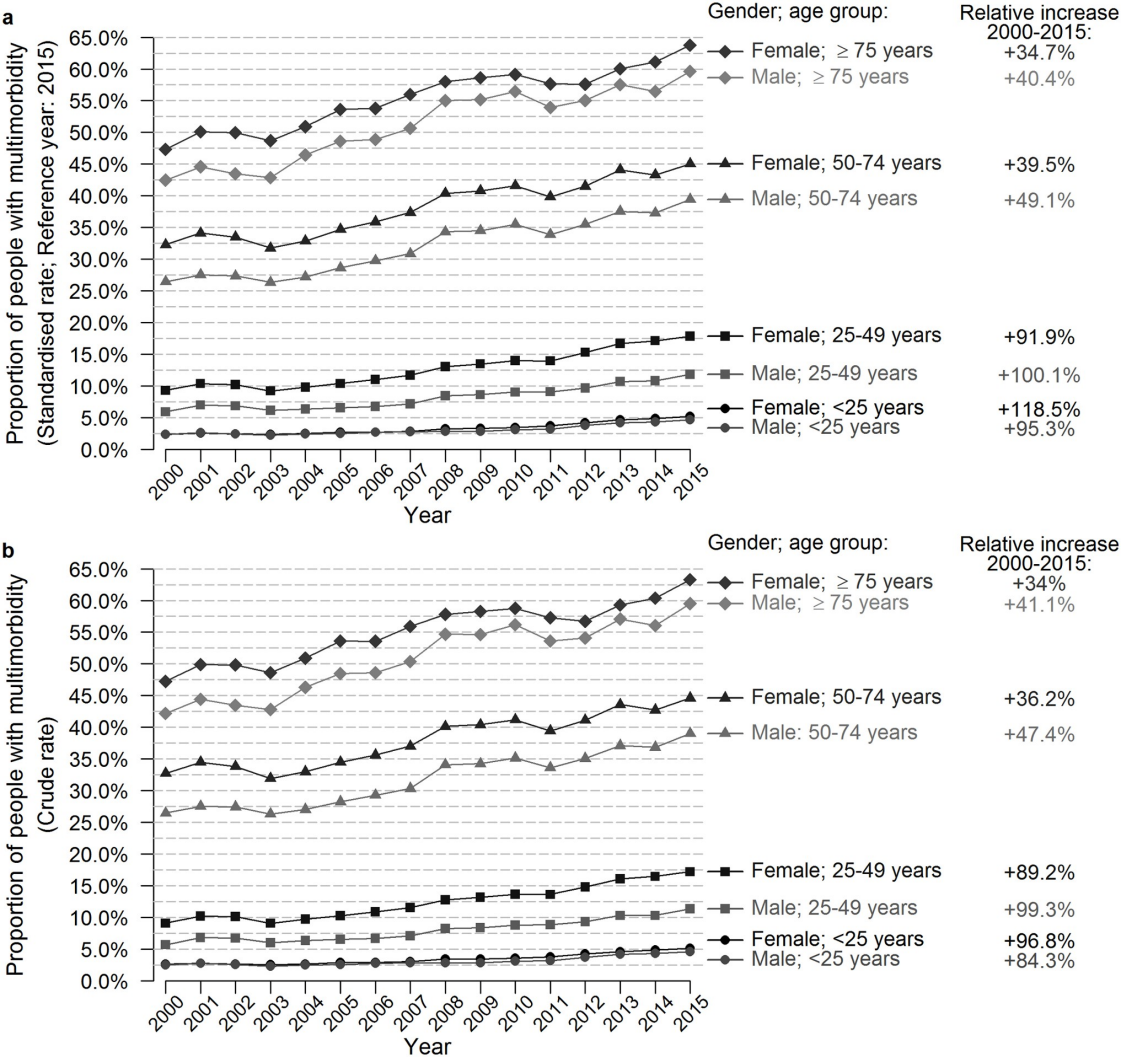
Evolution of the age- and sex-standardised prevalence rate of multimorbidity from 2000–2015.

Throughout the study period, the standardised prevalence rate of multimorbidity rose for both sexes and in all age groups. The largest relative increase was observed in the younger age groups (up to the age of 50 years), with the standardised prevalence rate of multimorbidity doubling between 2000 and 2015 (Fig 2a). Similar trends were found looking at the crude figures (Fig 2b). Trend analysis showed a stable increase in the standardised prevalence rate of multimorbidity for both males and females above the age of 50 and above 75 years (AAPC of 2.4% and 1.8% per year for females, and 2.9% and 2.3% per year for males, respectively) (Table 2). Young (0–25 years) females and males showed a modest but significant annual increase in the first period (APC of 2.0% per year between 2000–2006 and of 2.1% between 2000–2009, respectively) and a stronger significant increase afterwards (APC of 7.7% per year between 2006–2015 and 9.2% between 2009–2015, respectively). For females and males aged 25–49 years, there was an insignificant increase in the first period, but a significant increase of 5.7% per year between 2004–2015 and of 6.0% per year between 2005–2015, respectively.

**Table 2:** Joinpoint regression of the evolution of the age- and sex-standardised prevalence rate of multimorbidity between 2000 and 2015.

Group	SR in 2000–2015 (%)	Summary AAPC	Trend 1		Trend 2	
			Years	APC (95%CI)	Years	APC (95%CI)
***Total***	16.4–24.9	2.9 (2.5; 3.3)**				
***Females***	18.7–27.9	2.8 (2.4; 3.2)**				
<25 years	2.4–5.2	5.6 (4.7; 6.5)**	2000–2006	2.0 (-0.1; 4.1)*	2006–2015	7.7 (6.5; 8.9)**
25–49 years	9.3–17.9	4.7 (3.9; 5.4)**	2000–2004	0.4 (-3.5; 4.5)	2004–2015	5.7 (4.8; 6.6)**
50–74 years	62.3–45.0	2.4 (2.0; 2.9)**				
≥75 years	47.3–63.8	1.8 (1.5; 2.1)**				
***Males***	14.0–22.0	3.1 (2.6; 3.5)**				
<25 years	2.4–4.6	4.6 (3.5; 5.7)**	2000–2009	2.1 (0.5; 3.7)**	2009–2015	9.2 (6.0; 12.4)**
25–49 years	5.9–11.9	4.5 (3.6; 5.4)**	2000–2005	4.5 (-3.1; 4.5)	2005–2015	6.0 (4.6; 7.4)**
50–74 years	26.5–39.4	2.9 (2.4; 3.3)**				
≥75 years	42.5–59.6	2.3 (1.9; 2.8)**				

*p<0.10;

** p<0.05

SR: age- and sex-standardised prevalence rate; AAPC: Average annual percent change; APC: annual percent change; 95%CI: 95% confidence interval

**Fig 3:**
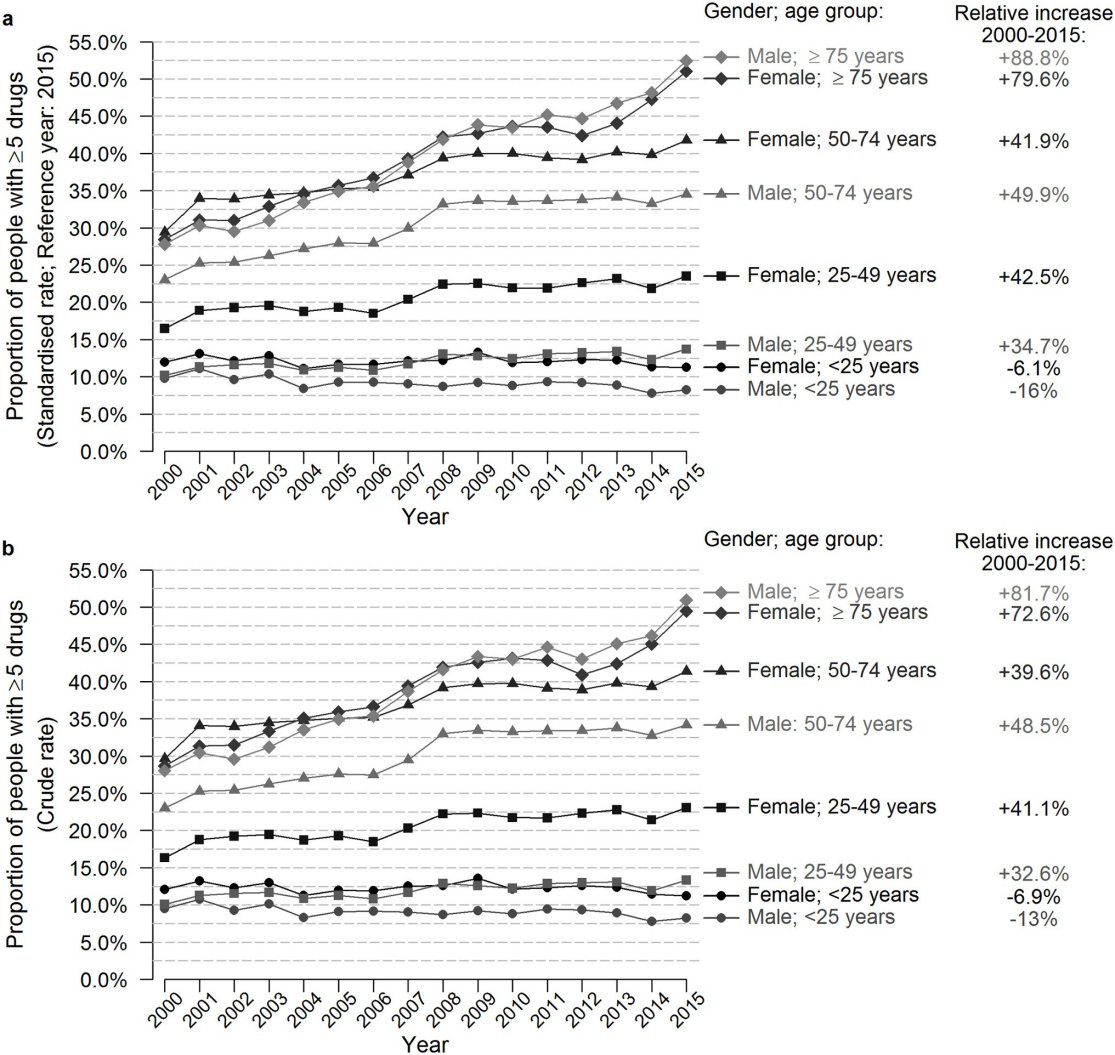
Evolution of the age- and sex-standardised and crude prevalence rates of polypharmacy (≥5 drugs) from 2000–2015.

The prevalence of polypharmacy showed a significant increase—both crude and standardized—between 2000 and 2015 for people aged 75 years or older, with an 89% and 80% relative increase for males and females, respectively (Fig 3a and 3b). The AAPC was 3.5% and 4.2% per year, respectively, with the highest increase for females of 75 years and older in the period 2013–2015 with an APC of 8.3%. Far more modest but still significant relative increases, of 50% for males and 42% for females, were found for people aged 50–74 years. In this age group, the AAPC was 1.8% for females; for males a significant increase (APC 4.0%) was found in the period 2000–2009 only (Table 3). Males and females aged 25–49 years had an AAPC of 1.6% and 1.9%, respectively, whereas no significant changes in polypharmacy were found for people aged below 25 years.

**Table 3:** Joinpoint regression of the evolution of the age- and sex-standardised prevalence rate of polypharmacy (≥5 drugs) between 2000 and 2015.

Group	SR in 2000–2015 (%)	Summary AAPC	Trend 1		Trend 2		Trend 3	
			Years	APC (95%CI)	Years	APC (95%CI)	Years	APC (95%CI)
**Total**	18.0–25.2	2.0 (1.6; 2.4)**						
***Females***	20.5–28.9	1.9 (1.4; 2.3)**						
<25 years	12.0–11.3	-0.3 (-0.9; 0.3)						
25–49 years	16.5–23.5	1.9 (1.4; 2.5)**						
50–74 years	294–41.8	1.8 (1.4; 2.3)**						
≥75 years	28.4–51.0	3.5 (3.0; 4.0)**	2000–2009	4.6 (4.0; 5.2)**	2009–2013	0.3 (-2.7; 3.4)	2013–2015	8.3 (1.9; 15.2)**
***Males***	15.4–21.5	2.1 (1.7; 2.6)**						
<25 years	9.8–8.2	-1.3 (-2.0; -0.6)**						
25–49 years	10.2–13.7	1.6 (1.0; 2.1)**						
50–74 years	23.0–34.5	2.7 (2.2; 3.3)**	2000–2009	4.0 (3.2; 4.8)**	2009–2015	0.5 (-0.9; 1.9)		
≥75 years	27.8–52.4	4.2 (3.7; 4.6)**						

** p<0.05

SR: age- and sex-standardised prevalence rate; AAPC: Average annual percent change; APC: annual percent change; 95%CI: 95% confidence interval

## Discussion

### Main findings

This study reports on the evolution of multimorbidity and polypharmacy in Flemish-Belgian primary care covering a 15-year period. In 2015 we found an overall prevalence of 22.7% for multimorbidity and 20% for polypharmacy. Both multimorbidity and polypharmacy were strongly related to higher age, although the absolute number for both multimorbidity and polypharmacy was the highest in the age group 50–74 years. For multimorbidity, we observed an increasing prevalence through the years for all age groups, with an even steeper slope for the younger age groups during the recent past. For polypharmacy, we observed a more moderate evolution for people aged less than 50 years as compared to those aged 75 and older.

### Context with previous findings

Multimorbidity rates are always difficult to compare, due to the large variation of methodological choices and populations studied [24, 25]. Nevertheless, the prevalence pattern in age and sex groups in 2015 in our study seems to be similar to that found in the UK [9]. Our results regarding the *evolution* of multimorbidity are more pronounced than those in other studies. Uijen *et al*. found a modest standardized increase of people with two or three chronic conditions, but a more pronounced increase of people with four or more chronic diseases in the Dutch population between 1995 and 2005 [17]. Another Dutch study [18] showed a modest standardized increase of 2.7% over a 7-year period for people aged 75 years and older, while a Swedish study [8] found stable prevalence rates of multiple severe symptoms/diseases among older people (over 77 years of age) between 2002 and 2011. However, these latter numbers were generated using self-reported diseases.

The rising prevalence of multimorbidity can be considered in the light of several factors. They include medical developments, such as improved diagnostics and better treatments, resulting in more frequent cures of acute diseases and less frequent or less serious adverse events, and hence longer survival after both acute and chronic illness. Other relevant global factors include the end of large-scale wars and the extreme improvement of living conditions [26].

The more pronounced increase of multimorbidity among people aged under 50 years in the second half of the study might be the result of increasingly efficient coding of diseases. For patients aged over 50 this effect would be smaller and hence not result in a significantly different trend, because many of them already were over the threshold of two chronic conditions.

The prevalence rates of polypharmacy found in our study were comparable to other studies from Western societies. The latter reported prevalence rates of polypharmacy between 27% and 59% in primary care patients aged 65 years and older [27] or community-dwelling elderly of the same age living in the USA [28]. A recent study from the UK reported the proportion of adults with polypharmacy had doubled to 20.8% between 1995 and 2000 [16]. We know that our database might have an under-registration of prescribed medication: medication prescribed by medical hospital specialists as well as medication prescribed during home visits might be incomplete [29].

The plateau in the polypharmacy trend for females ≥75 years, might be related to a relative increase in new young GPs in this period, who do less home consultations which compared to their older GP-colleagues.

### Strengths and limitations

The analyses for this study were performed using a large database; for 2015, we had information on over 150,000 individual patients. In 2014, the estimated practice population in the Intego database represented 2.3% of the Flemish-Belgian population. Moreover, the Intego population is representative of the Flemish-Belgian population in terms of age and sex [19].

General practices have to pass three quality criteria before being accepted as participants in Intego [19]. This results in a reliable morbidity database containing routinely collected data in primary care, representing daily clinical practice. External validation of the Intego database has been examined by means of national and international comparisons [19]. However, previous analyses have shown that the registration of medication is not always complete [29]. This may result in an underestimation of polypharmacy rates, but we do not expect this to affect the trends reported.

### Implications for clinical practice

Primary care for patients with multimorbidity and polypharmacy is complex—both for patients and health care professionals—and patients are prone to safety incidents [30]. It can be expected that the trend will continue of an increasing number of patients having to deal with multimorbidity and polypharmacy. This underlines the need for care innovations for this group of complex patients. It is increasingly accepted that understanding and including patients’ preferences is of the utmost importance in optimising care for patients with multimorbidity [31]. Attempts to meet the patients’ needs in case of multimorbidity are e.g. minimally disruptive medicine [32, 33] and the Ariadne principles, which offer guidance on how to handle multimorbidity in primary care consultations [14]. Both models acknowledge the importance of the patient’s role as well as patient-physician communication in care. Taking care of patients with multimorbidity requires GPs and other caregivers who are capable of delivering goal-oriented care for those patients and proactive care for the prevention of chronic diseases.

### Implications for future research and health policy

The epidemiology of chronic disease, multimorbidity and polypharmacy is dynamic. Reliable and up-to-date analyses are necessary to guide health policy, physicians and medical guideline development. Furthermore, the authors of care models focusing on patients with multimorbidity give indications of how to use disease models or principles in daily practice, but the training of doctors in the management of patients with multimorbidity seems to be hardly evaluated [34]. The current evidence of interventions developed for the care of people with multimorbidity and polypharmacy is ambiguous [35–37]. It is clear, however, that both patients and health care professionals feel an urgent need for care coordination and harmonization of treatments and other medical procedures, using interdisciplinary expertise and patients’ preferences and goal setting [14, 38]. In order to reach better care for patients with multimorbidity and polypharmacy, both concepts should be part of the educational programmes for physicians, pharmacists and other health care workers to train interprofessional collaboration [39, 40].

## Conclusion

For all adult age groups, multimorbidity and polypharmacy are frequent, dynamic over time and increasing. This situation demands both epidemiological and interventional studies to improve the management of the resulting complex care.

## Supporting information

S1 Data (ZIP)

S1 Fig Participation of practices during study period. (DOCX)

S1 Table Number of people in the yearly contact group and the practice population in Intego between 2000 and 2015. (DOCX)

S2 Table list of chronic diseases. (DOCX)

S3 Table Logistic regression (age groups considered as a continuous variable to test for the trend), with multimorbidity in 2015 as the outcome. (DOCX)
