# Supplementary material for: Trends in multimorbidity and polypharmacy in the Flemish-Belgian population between 2000 and 2015
Source: PLoS One. 2019 Feb 12;14(2):e0212046. doi: 10.1371/journal.pone.0212046 (PMC6372187; doi:10.1371/journal.pone.0212046)
Supplement: S1 Fig — (DOCX) [file pone.0212046.s002.docx]

S1 Fig.: participation of practices during study period
