# Supplementary material for: Trends in multimorbidity and polypharmacy in the Flemish-Belgian population between 2000 and 2015
Source: PLoS One. 2019 Feb 12;14(2):e0212046. doi: 10.1371/journal.pone.0212046 (PMC6372187; doi:10.1371/journal.pone.0212046)
Supplement: S1 Table — (DOCX) [file pone.0212046.s003.docx]

S1 Table. Number of people in the yearly contact group and the practice population in Intego between 2000 and 2015

| Year | 2000 | 2001 | 2002 | 2003 | 2004 | 2005 | 2006 | 2007 |
| --- | --- | --- | --- | --- | --- | --- | --- | --- |
| Yearly contact group | 95.932 | 90.973 | 106.664 | 125.202 | 120.962 | 128.251 | 133.931 | 132.322 |
| Practice population | 122.340 | 115.328 | 135.093 | 158.878 | 161.327 | 171.397 | 178.843 | 176.852 |
| Year | 2008 | 2009 | 2010 | 2011 | 2012 | 2013 | 2014 | 2015 |
| Yearly contact group | 134.733 | 140.259 | 140.126 | 151.971 | 127.717 | 130.398 | 131.651 | 123.261 |
| Practice population | 168.311 | 175.534 | 175.196 | 186.829 | 157.130 | 161.105 | 162.727 | 152.238 |
