# Supplementary material for: Trends in multimorbidity and polypharmacy in the Flemish-Belgian population between 2000 and 2015
Source: PLoS One. 2019 Feb 12;14(2):e0212046. doi: 10.1371/journal.pone.0212046 (PMC6372187; doi:10.1371/journal.pone.0212046)
Supplement: S2 Table — (DOCX) [file pone.0212046.s004.docx]

| S2 Table: list of chronic diseases | |
| --- | --- |
| **ICPC-2 Code** | **ICPC-2**  **Title** |
| A79 | Malignancy NOS |
| A90 | Congenital anomaly OS/multiple |
| B72 | Hodgkin's disease/lymphoma |
| B73 | Leukaemia |
| B74 | Malignant neoplasm blood other |
| B78 | Hereditary haemolytic anaemia |
| B83 | Purpura/coagulation defect |
| B90 | HIV-infection/aids |
| D74 | Malignant neoplasm stomach |
| D75 | Malignant neoplasm colon/rectum |
| D76 | Malignant neoplasm pancreas |
| D77 | Malig. neoplasm digest other/NOS |
| F83 | Retinopathy |
| F84 | Macular degeneration |
| F94 | Blindness |
| H83 | Otosclerosis |
| H84 | Presbyacusis |
| H86 | Deafness |
| K74 | Ischaemic heart disease w. angina |
| K75 | Acute myocardial infarction |
| K76 | Ischaemic heart disease w/o angina |
| K77 | Heart failure |
| K82 | Pulmonary heart disease |
| K86 | Hypertension uncomplicated |
| K87 | Hypertension complicated |
| K90 | Stroke/cerebrovascular accident |
| K91 | Cerebrovascular disease |
| K92 | Atherosclerosis/PVD |
| K93 | Pulmonary embolism |
| K94 | Phlebitis/thrombophlebitis |
| L84 | Back syndrome w/o radiating pain |
| L85 | Acquired deformity of spine |
| L88 | Reumatoãde arthritis |
| L89 | Osteoarthrosis of hip |
| L90 | Osteoarthrosis of knee |
| L91 | Osteoarthrosis other |
| L95 | Osteoporosis |
| L98 | Acquired deformity of limb |
| N70 | Poliomyelitis |
| N74 | Malignant neoplasm nervous system |
| N85 | Congenital anomaly neurological |
| N86 | Multiple sclerosis |
| N87 | Parkinsonism |
| N88 | Epilepsy |
| N89 | Migraine |
| N90 | Cluster headache |
| N92 | Trigeminal neuralgia |
| P28 | Limited function/disability (p) |
| P70 | Dementia |
| P71 | Organic psychosis other |
| P72 | Schizophrenia |
| P73 | Affective psychosis |
| P74 | Anxiety disorder/anxiety state |
| P75 | Somatization disorder |
| P76 | Depressive disorder |
| P77 | Suicide/suicide attempt |
| P79 | Phobia/compulsive disorder |
| P80 | Personality disorder |
| P85 | Mental retardation |
| P98 | Psychosis NOS/other |
| R79 | Chronic bronchitis |
| R84 | Malignant neoplasm bronchus/lung |
| R85 | Malinant neoplasm respiratory, other |
| R95 | Chronic obstructive pulmonary disease |
| R96 | Asthma |
| S77 | Malignant neoplasm of skin |
| S87 | Dermatitis/atopic eczema |
| S91 | Psoriasis |
| S97 | Chronic ulcer skin |
| T71 | Malignant neoplasm thyroid |
| T80 | Congenital anom endocrine/metab. |
| T85 | Hyperthyroidism/thyrotoxicosis |
| T86 | Hypothyroidism/myxoedema |
| T89 | Diabetes insulin dependent |
| T90 | Diabetes non-insulin dependent |
| T92 | Gout |
| T93 | Lipid disorder |
| T99 | Endocrine/metab/nutrit. dis. other |
| U04 | Incontinence urine |
| U75 | Malignant neoplasm of kidney |
| U76 | Malignant neoplasm of bladder |
| U77 | Malignant neoplasm urinary other |
| U85 | Congenital anomaly urinary tract |
| U88 | Glomerulonephritis/nephrosis |
| W72 | Malignant neoplasm relate to preg. |
| X75 | Malignant neoplasm cervix |
| X76 | Malignant neoplasm breast female |
| X77 | Malignant neoplasm genital other (f) |
| Y77 | Malignant neoplasm prostate |
| Y78 | Malign neoplasm male genital other |
| Y85 | Benign prostatic hypertrophy |
