# Supplementary material for: Trends in multimorbidity and polypharmacy in the Flemish-Belgian population between 2000 and 2015
Source: PLoS One. 2019 Feb 12;14(2):e0212046. doi: 10.1371/journal.pone.0212046 (PMC6372187; doi:10.1371/journal.pone.0212046)
Supplement: S3 Table — (DOCX) [file pone.0212046.s005.docx]

S3 Table: Logistic regression (age groups considered as a continuous variable to test for the trend), with multimorbidity in 2015 as the outcome

|  | Univariate analysis | | Multivariable analysis | | Multivariable analysis | |
| --- | --- | --- | --- | --- | --- | --- |
|  | OR (95%CI) | p-value | OR (95%CI) | p-value | OR (95%CI) | p-value |
| Age groups, continuous | 3.41 (3.35; 3.46) | <0.001 | 3.40 (3.34; 3.45) | <0.001 | 3.29 (3.22; 3.36) | <0.001 |
| Male (vs female) | 0.74 (0.73; 0.76) | <0.001 | 0.76 (0.74; 0.78) | <0.001 | 0.68 (0.64; 0.72) | <0.001 |
| Interaction age groups*gender |  |  |  |  | 1.08 (1.04; 1.11) | <0.001 |
